# Diversity of Cultivable Protease-Producing Bacteria in Laizhou Bay Sediments, Bohai Sea, China

**DOI:** 10.3389/fmicb.2017.00405

**Published:** 2017-03-16

**Authors:** Yan Li, Chaoya Wu, Mingyang Zhou, En Tao Wang, Zhenpeng Zhang, Wei Liu, Jicai Ning, Zhihong Xie

**Affiliations:** ^1^Key Laboratory of Coastal Biology and Utilization, Yantai Institute of Coastal Zone Research, Chinese Academy of SciencesYantai, China; ^2^School of Chemical and Biological Engineering, Lanzhou Jiaotong UniversityLanzhou, China; ^3^School of Chemistry and Pharmaceutical Engineering, Qilu University of TechnologyJinan, China; ^4^Departamento de Microbiología, Escuela Nacional de Ciencias Biológicas, Instituto Politécnico NacionalMexico City, Mexico

**Keywords:** protease-producing bacteria, diversity, Laizhou Bay, inhibition test, extracellular protease diversity

## Abstract

Protease-producing bacteria are widespread in ocean sediments and play important roles in degrading sedimentary nitrogenous organic materials. However, the diversity of the bacteria and the proteases involved in such processes remain largely unknown especially for communities in enclosed sea bays. Here, we investigated the diversity of the extracellular protease-producing bacteria and their protease types in Laizhou Bay. A total of 121 bacterial isolates were obtained from sediment samples in 7 sites and their protease types were characterized. The abundance of cultivable protease-producing bacteria was about 10^4^ CFU g^−1^ of sediment. Phylogenetic analysis based on 16S rRNA gene sequences suggest that the isolates belonged to 17 genera from 4 phyla including *Firmicutes, Actinobacteria, Proteobacteria* and *Bacteroidetes*, and mainly dominated by the genera *Pseudoalteromonas* (40.5%), *Bacillus* (36.3%), and *Photobacterium* (5.8%). The diversity and community structure varied among different sampling sites but no significant correlation was observed with soil sediment's characteristics. Enzyme activity and inhibition tests further revealed that all isolates secreted proteases that were inhibited by serine and/or metalloprotease inhibitors, and a smaller proportion was inhibited by inhibitors of cysteine and/or aspartic proteases. Furthermore, all isolates effectively degraded casein and/or gelatin with only a few that could hydrolyze elastin, suggesting that the bacteria were producing different kinds of serine proteases or metalloproteases. This study provided novel insights on the community structure of cultivable protease-producing bacteria near the Yellow River estuary of an enclosed sea bay.

## Introduction

Polymeric and particulate materials, which carry abundant organic nitrogen (OrgN), are the main nitrogen sources in marine environments. These materials usually precipitate in ocean sediments and are thus, involved in the global nitrogen biogeochemical cycle (Thamdrup and Dalsgaard, 2008). In the nitrogen cycle, the particulate OrgN decomposes into dissolved OrgN before it undergoes ammonification, nitrification and denitrification resulting to its release into the atmosphere as nitrogen gas. These processes are mainly carried out by bacteria that produce degradation enzymes (Brunnegård et al., 2004; Hunter et al., 2006). Since proteins comprise the main component of biomass of marine organisms (Thamdrup and Dalsgaard, 2008; Lloyd et al., 2013; Moore et al., 2014), protease-producing bacteria are considered as the main degraders of organic nitrogen in the marine environment (Chen et al., 2003; Zhao et al., 2012). These protease-producing bacteria usually secrete extracellular proteases that degrade protein materials, for example, by hydrolyzing the OrgN into peptides and amino acids, with the latter easily taken up by bacteria for subsequent catabolism (Zhao et al., 2012). Although, protease-producing bacteria play key roles on ecological and biochemical cycles in marine sediments, few studies have been done to understand the diversity of these species and of their extracellular proteases. Nineteen protease-producing bacteria isolated from the sub-Antarctic sediments were classified into five genera, namely *Proteobacteria* (*Pseudoalteromonas, Shewanella, Colwellia*), *Firmicutes* (*Planococcus*), and *Bacteroidetes* (*Olleya*), with *Pseudoalteromonas* as the dominant one (Olivera et al., 2007). Among 98 strains isolated from Southern Okinawa Trough, 30 of them possessed protease-producing abilities, which belong to the genus of *Bacillus, Cobetia, Halomonas, Pseudomonas, Psychrobacter, Myroides, Planococcus, Sporosarcina*, and *Wangia*. Among them, strains belong to *Bacillus* and *Psychrobacter* showed excellent abilities to produce neutral, acidic, and alkaline proteases at low temperature conditions (Dang et al., 2009). Out of the 78 protease-producing bacteria isolated from the sediments of the deep South China Sea, 77 isolates belonged to *Gammaproteobacteria* under *Alteromonas, Pseudoalteromonas, Marinobacter, Idiomarina, Halomonas, Vibrio, Shewanella, Pseudomonas*, and *Rheinheimera*; while a single isolate belonged to *Firmicutes* (*Bacillus*), and dominated mostly by *Alteromonas* and *Pseudoalteromonas* accounting for 34.6 and 28.2% of the total relative abundance, respectively (Zhou et al., 2009). Strains with extracellular proteolytic activities from the coastal sediments of King George Island, Antarctica showed high diversity, with the 105 isolates belonging to four major phyla *Actinobacteria, Firmicutes, Bacteroidetes*, and *Proteobacteria* and dominated by *Bacillus, Flavobacterium*, and *Lacinutrix* (Zhou et al., 2013). Nearly all of the extracellular proteases secreted by these bacteria were serine and/or metalloproteases (Zhou et al., 2009, 2013). A similar community profile was observed in marine sediments of the eutrophied Jiaozhou Bay, China, where 69 protease-producing bacteria belonging to 9 genera from three phyla were isolated, including *Bacteroidetes, Firmicutes*, and *Proteobacteria*, in which *Photobacterium, Bacillus* and *Vibrio* were the dominant groups, also mainly producing serine and/or metalloproteases with relatively low proportions of cysteine proteases (Zhang et al., 2015).

Laizhou Bay, located between 37.05° ~37.80° N and 118.9° ~120.35° E (WGS84 reference system) is a typical semi-enclosed inner sea, located in the southern coast of Bohai Sea, North China, occupying around 10% of the total area of the Bohai Sea (Zhang et al., 2012; Wang et al., 2015). The bay is a traditional fishing ground of China. However, increased intensities in fishing activity, industrial discharges, agricultural and domestic sewage, and human disturbances in the last decades resulted to deteriorated water quality, decreased biomass and fish biodiversity in the Bay (Zhang et al., 2012; Jin et al., 2013; Wang et al., 2015). With the corresponding water pollution, the associated microbial community in this aquatic ecosystem, especially the degrading bacteria, are also expected to vary. Further, the population structure of protease producing bacteria in disturbed ecosystems like that of Bohai Sea, would significantly differ compared to Antarctic sediment samples which are less disturbed by human activities. However, the protease-producing bacteria and their extracellular protease in the sediments of Laizhou Bay have not yet been investigated. This study then aims to uncover the diversity of protease-producing bacterial community in Laizhou Bay and characterize their extracellular proteases using inhibitor tests.

## Materials and methods

### Sediment collection and physiochemical characteristics

**Figure 1:**
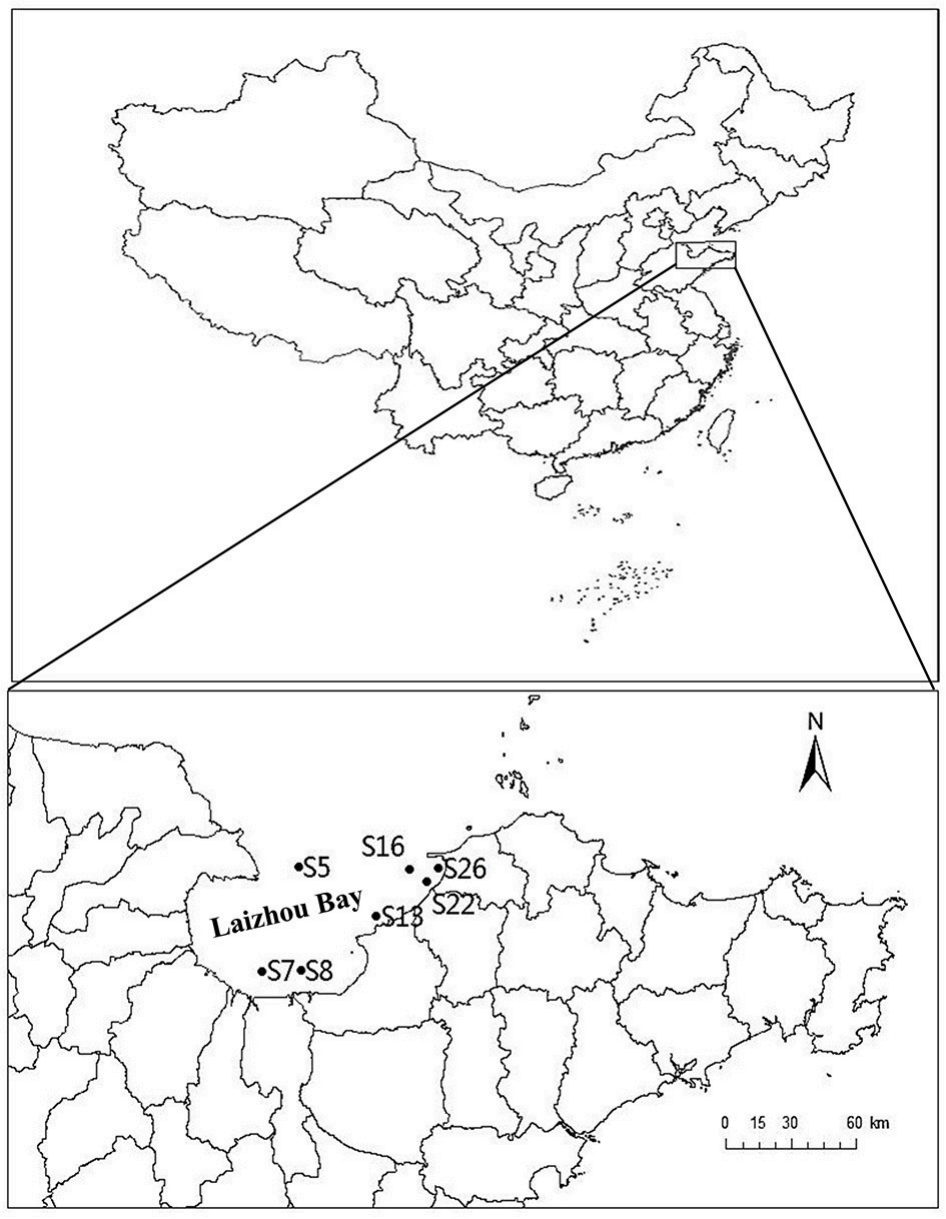
**Map of the Laizhou Bay, China showing the sampling sites (•)**. The corresponding position of the Laizhou Bay in China is shown in the inset. The two maps were created using DIVA-GIS software (http://www.diva-gis.org), and the sampling sites were added according to GPS records.

A total of seven sediment samples were collected from different sites of Laizhou Bay (Figure 1 and Table 1) using a 0.05 m^2^ stainless steel Gray O'Hara box corner. Collection was carried out on September 2013. Six of the stations were near the shore and only Station 5 was located offshore. Triplicate surface sediment sub-core samples (0–5 cm depth) were collected using sterilized 60 ml syringes (without luer end) and then transferred to airtight sterile plastic bags at 4°C until analysis in the laboratory (Zhou et al., 2009). For physiochemical analyses, samples were collected with the same procedure and stored in sterilized plastic bags at −20°C during the cruise and were transferred to −80°C upon arrival in the laboratory. Surface temperature and pH of sediment samples were detected *in situ* using a pH meter. Organic carbon and nitrogen concentrations were determined using a PE 2400 series II CHNS/O analyzer (Perkin Elmer, USA).

**Table 1:** **Characteristics of the sampling stations and the distribution of different genera in these stations**.

**Properties**		**Stations**						
		**S5**	**S7**	**S8**	**S13**	**S16**	**S22**	**S26**
GPS		N37.65 E119.55	N37.25 E119.35	N37.25 E119.55	N37.45 E119.95	N37.63 E120.13	N37.58 E120.22	N37.63 E120.28
**CHARACTERISTICS FOR SEDIMENT SAMPLES**								
Depth (m)		11.4	7.0	8.6	14.4	13.8	10.5	8.2
Temperature (°C)		20.02	20.83	21.67	22.47	22.38	22.11	24.26
pH		8.15	8.28	8.17	8.18	8.14	8.16	8.26
OrgC (%)		1.20	1.82	0.85	0.95	1.27	1.01	0.29
OrgN (%)		0.08	0.10	0.01	0.01	0.08	0.07	0.02
C/N^a^		15.00	18.02	85.00	95.00	15.88	14.43	14.50
**GENERA DISTRIBUTION**								
*Firmicutes*	*Bacillus*	0	10	3	0	2	14	15
	*Jeotgalibacillus*	0	0	0	0	0	1	0
	*Planococcus*	0	0	0	0	0	0	1
	*Oceanobacillus*	0	0	0	0	0	0	2
	*Halobacillus*	0	0	1	0	0	3	1
*Proteobacteria*	*Pseudoalteromonas*	11	6	13	3	11	4	1
	*Sulfitobacter*	1	0	0	0	0	0	0
	*Marinobacter*	2	0	0	0	0	0	0
	*Halomonas*	0	0	0	1	0	0	0
	*Rheinheimera*	0	0	0	0	1	0	0
	*Celeribacter*	0	0	0	0	0	0	1
	*Photobacterium*	0	1	0	6	0	0	0
	*Ruegeria*	0	2	0	0	0	0	0
	*Alcanivorax*	0	0	0	0	0	1	0
*Actinobacteria*	*Micrococcus*	1	0	0	0	0	0	0
	*Nocardioides*	0	0	0	1	0	0	0
*Bacteroidetes*	*Salegentibacter*	1	0	0	0	0	0	0
Total strain number (121)		16	19	17	11	14	23	21
**DIVERSITY INDEX**								
H'Shannon–Wiener (*H'*)		1.04	1.09	0.68	1.12	0.66	1.14	1.04
Simpson (*D*)		0.50	0.61	0.38	0.61	0.36	0.58	0.47
Pielou (*J*)		0.64	0.79	0.62	0.81	0.60	0.71	0.58

a *C/N is the abbreviation of OrgC/OrgN*.

### Screening of protease-producing bacteria from sediment samples

Protease-producing bacteria were screened as previously described (Zhou et al., 2009; Zhang et al., 2015). Briefly, 1 g (fresh weight, triplicate samples collected from the same station were weighed equally before mixing) of each sediment sample was serially diluted to 10^−6^ with artificial sea water. Aliquots of 100 μl of the serially diluted samples (10^−1^–10^−6^) were separately spread on the screening plates composed of 0.2% (w/v) yeast extract, 0.3% (w/v) casein, 0.5% (w/v) gelatin, 1.5% (w/v) agar powder, in 1 L artificial seawater at pH 8.0. All plates with inoculum were incubated at 25°C until colonies with clear hydrolysis zones were detected. Colonies with different morphological characteristics (e.g., colony color, size and surface polysaccharides) were selected and further purified by repeated streaking on the same plate until uniform or pure colonies were observed. Pure cultures were preserved at −80°C in 20% (v/v) glycerol.

### 16S rRNA gene amplification and phylogeny

Each pure isolate was first incubated in the liquid medium (screening media without agar) at 25°C with shaking of 200 rpm. The biomass of each isolate was collected through centrifugation and the genomic DNA was extracted using a TIANGEN genomic DNA extraction kit (TIANGEN, China) for bacteria, following the manufacturer's instructions. Both DNA quality and quantity were determined in Nano OD2000C (Thermo). The 16S rRNA gene of all the isolates were amplified and sequenced from genomic DNA using the universal primer pair 27F-1492R following the PCR conditions described by Engel et al. (2004). Samples were sent to the Beijing AuGCT DNA-SYN Biotechnology Co., Ltd for sequencing using the Sanger method (Sanger et al., 1977). Identification of the sequences generated in this study was carried out by searching for their most similar sequences in the NCBI GenBank using the BLASTn approach. Then, a 16S rRNA gene phylogenetic tree was reconstructed using the neighbor-joining method (Saitou and Nei, 1987) with Kimura's two-parameter model in MEGA version 5.05 (Tamura et al., 2011). The topology of phylogenetic trees was evaluated by bootstrap method with 1,000 replications, also in MEGA version 5.05. All sequences were deposited in GenBank database.

**Figure 2:**
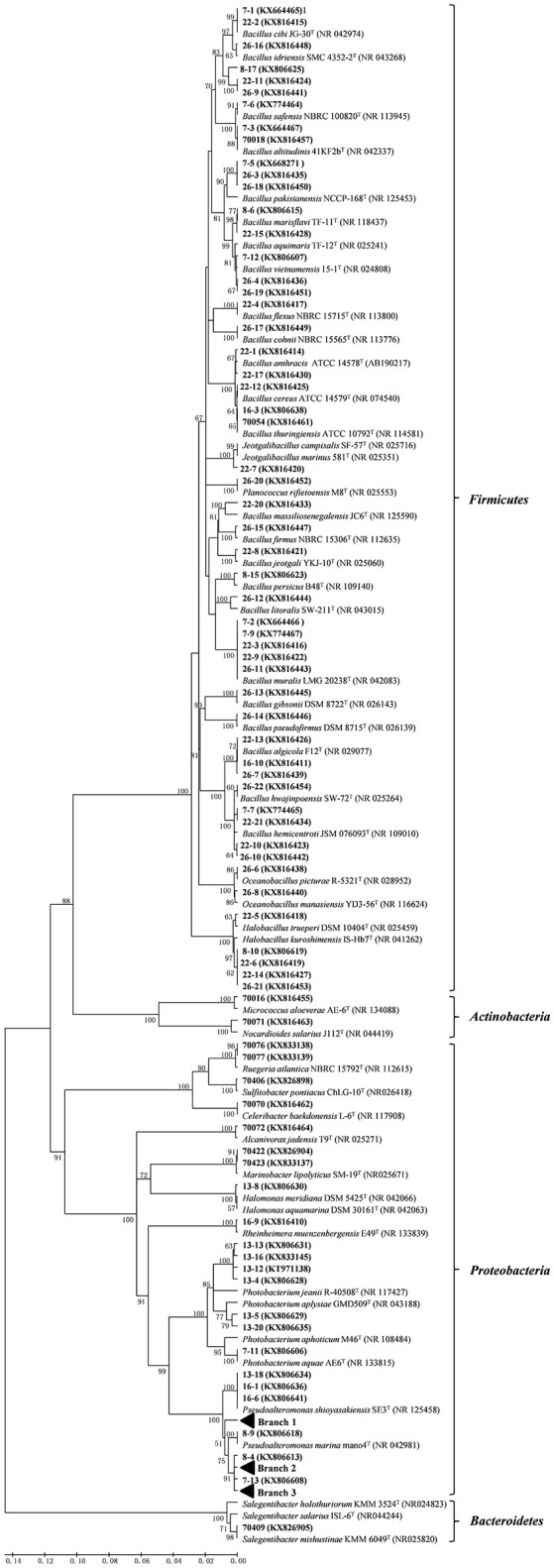
**Phylogenetic tree of the protease-producing bacteria isolated from Laizhou Bay, China based on 16S rRNA gene sequences**. Taxa and GenBank accession numbers in boldface were generated in this study. The tree was constructed by neighbor-joining method using MEGA version 5.05. Only bootstrap values greater than 50% are presented in the nodes. The scale bar represents 2% nucleotide substitution.

Due to the limitation in the resolution of the 16S rRNA gene and since some species may have identical 16S rRNA gene sequences (Figure 2), not all sequences were identified at the species level. Alpha diversity of the community based on genera in each sample was investigated three indices that have specific targets, namely the Shannon–Wiener index (*H*′), which explains the species richness of a sample site, the Simpson index (*D*) that shows the dominance species, and the Pielou index (*J*), which indicates species evenness in a community (Hill et al., 2003). These diversity indices were estimated from each sample using the Vegan package (version 1.17-4) implemented in the R environment (version3.1.2; http://www.r-project.org/) (R-Core Team, 2014).

### Hydrolysis of casein, elastin, and gelatin by bacteria extracellular proteases

Solid basic media (0.2% (w/v) yeast extract, 0.5% (w/v) gelatin, 1.5% (w/v) agar powder, artificial seawater, pH 8.0) supplemented with 0.5% (w/v) of casein, 0.5% (w/v) gelatin or 0.5% (w/v) elastin powder (Zhou et al., 2009), were used in this particular analysis. Isolates were inoculated with sterilized tooth stick on the plates in the medium and incubated at 25°C for 3 days. The diameter of colony and the hydrolyzed zone were measured for each strain, and a ratio of the hydrolytic zone diameter vs. the colony diameter (hydrolytic zone/colony, H/C) was calculated as a proxy for enzyme activity for each substrate (Zhou et al., 2009).

### Inhibition ratio (%) of different inhibitors on the protease activity

Each protease-producing isolate was cultivated in the liquid screening medium (screening media without agar), and incubated at 25°C with shaking of 200 rpm for 3 days. The culture was centrifuged at 12,000 rpm for 2 min to collect the supernatant (Chen et al., 2003), which was subsequently used to measure the protease activity as previously described (Chen et al., 2003). In brief, 1 ml of the supernatants were diluted with 1 ml of 2.0% (w/v) casein in 50 mM Tris-HCl (pH 8.0) and incubated with the same volume of 1.0 mM phenylmethylsulfonyl fluoride (PMSF, Sigma; serine protease inhibitor), 1.0 mM 1, 10-phenanthroline (OP, Sigma; metalloprotease inhibitor), 0.1 mM E-64 (Merk; cysteine protease inhibitor) and 0.1 mM pepstatin A (P-A, Sigma; aspartic protease inhibitor) at 40°C for 20 min. After incubation, the reaction was stopped by the addition of 2 ml of 0.4 M trichloroacetic acid and incubated at 40°C for 10 min. Then, 1 ml of supernatant was neutralized with 5 ml of 0.4 M sodium carbonate and incubated with 1 ml of Folin–Ciocalteu's reagent solution (Sigma) at 40°C for 20 min. Finally, the protease activity was separately measured at 660 nm for each sample (Chen et al., 2003; Zhou et al., 2009; Zhang et al., 2015). One unit of enzyme activity was defined as the formation of 1 μmole tyrosine in 1 min. The protease activity of the sample without any inhibitor, characterized by 100% degradation, was designated as the control. The inhibition ratio (%) was determined as the result of control activity (without any inhibitor) minus the relative activity of a sample (Zhou et al., 2009; Zhang et al., 2015).

## Results

### Station location and sample characteristics

All the sampled sediments had slight alkaline pH values, which varied from 8.15 to 8.28 (Table 1). The water depths ranged from 7.0 to 14.4 m. The contents of OrgC in the sediments ranged from 0.29 to 1.82% (w/w) and OrgN from 0.01 to 0.10% (w/w). The highest values for both OrgC and OrgN were observed in station S7. However, the highest C/N ratio (95.00) was found in station S13 and the lowest (14.13) was in station S22.

### Quantification and isolation of protease-producing bacteria

Colonies with different colors, morphologies and sizes appeared on the screening plates inoculated with 10^−1^–10^−4^ dilutions after incubating at 25°C for 1–5 days. Colony counts in the plates indicated that the abundance of cultivated bacteria in each sample reached around 10^4^ CFU (colony forming unit) per gram of sediment samples, and nearly 60% colonies exhibited formation of hydrolytic zones in all the samples. Although OrgC and OrgN contents varied among the stations, no obvious correlation between the abundance of protease-producing bacteria and OrgC/OrgN contents was observed. One hundred twenty-one hydrolytic colonies were finally selected for subsequent analysis.

### Diversity of the protease-producing bacteria isolated from sediments

The 16S rRNA gene sequences (>1,400 bp) obtained from all 121 isolates were classified into 17 genera (Table 1). Except for a single isolate (70409) from station S5 belonging to *Salegentibacter* in the phylum *Bacteroidetes*, and two isolates belonging to *Micrococcus* (70016 also from station S5) and *Nocardioides* (70071 from station S13) in the phylum of *Actinobacteria*, the rest of the isolates were classified to 14 genera within phyla *Firmicutes* and *Proteobacteria*. These include *Bacillus, Jeotgalibacillus, Halobacillus, Planococcus*, and *Oceanobacillus* in *Firmicutes*; and *Pseudoalteromonas, Photobacterium, Halomonas, Rheinheimera, Alcanivorax, Celeribacter, Sulfitobacter, Marinobacter* and *Ruegeria* in *Proteobacteria*. *Bacillus* (36.4%), *Pseudoalteromonas* (40.5%) and *Photobacterium* (5.8%) were the predominant genera while *Jeotgalibacillus, Planococcus, Halomonas, Rheinheimera, Celeribacter, Salegentibacter, Micrococcus, Nocardioides, Alcanivorax* and *Sulfitobacter* were represented only by one isolate each, in total (10 isolates) corresponding to 8.3% of the total isolates. *Pseudoalteromonas* was isolated in all stations but most dominant in stations S5, S8, and S16 (Table 1). *Bacillus* was found in 5 stations and dominated in S7, S22, and S26 (Table 1). *Bacillus* and *Pseudoalteromonas* were the most abundant groups (93/121), both accounting for 76.9% of the cultivated protease-producing bacteria in Laizhou Bay sediments. Furthermore, the protease-producing bacteria isolated from S26 belonged to 6 genera, which showed higher diversity than those isolated from other sites. Only three genera were identified from S8 and S16, representing the least diverse community among the samples (Table S1).

As shown in Table 1, out of the 49 *Pseudoalteromonas* isolates, 22 isolates from 5 stations formed Branch 1 in Figure 2 and Figure S1, with 100% sequence similarity with *Pseudoalteromonas lipolytica* LMEB39^T^, a strain isolated from seawater of Yangtze River estuary in China. Nine *Pseudoalteromonas* isolates (Branch 2 from 4 stations) (Figure 2 and Figure S2) clustered with *Pseudoalteromonas spiralis* Te-2-2^T^ and *Pseudoalteromonas tetraodonis* IAM 14160^T^ with 99.7~99.9% similarity. Twelve isolates (from two sites) on the other hand formed Branch 3 (Figure 2 and Figure S3) that was closest to *Pseudoalteromonas hodoensis* H7^T^ and *Pseudoalteromonas atlantica* NBRC 103033^T^ (99.0 ~ 100%). Three isolates (13-18, 16-1, and 16-6) were identical to *Pseudoalteronomas shioyasakiensis* SE3^T^, and strains 8-9 with *Pseudoalteromonas marina* mano4^T^. Among the seven *Photobacterium* isolates, except isolate 7-11 that shared high similarity with *Photobacterium aquae* AE6^T^, the other two clusters with four (13-4, 13-12, 13-13, and 13-16) and two (13-5, 13-20) isolates, respectively could be potential novel species, since they had low similarities (95.7–97.1% similarities aligned on EzTaxon website) with their closest related species *Photobacterium jeanii* R-40508^T^ and *Photobacterium aplysiae* GMD509^T^, respectively (Figure 2). High diversity was detected among the 44 isolates of *Bacillus* (Table 1 and Figure 2) and most of them showed high similarities (>99%) in 16S rRNA gene sequences with known species. However, the branches included three isolates (8-17, 22-11, and 26-9) that showed low similarities (96.42–97.27%) with all recognized *Bacillus* species and may represent novel taxa needing further taxonomic studies (Figure 2). For the less abundant groups, all the isolates were highly similar or identical with the 16S rRNA gene sequences of some known or established species.

Shannon–Weiner (*H*′) index was highest in station S22 (1.14), followed by station S13 (1.12) and S7 (1.09) (Table 1). The lowest (0.66) was observed in station S16 where only three genera were found. The highest Simpson index (*D*) value (0.61) was found in stations S7 and S13, followed by stations S22 (0.58) and S5 (0.50). The lowest value (0.66) was observed in S16 (0.36). Pielou (*J*) ranged from 0.58 in the station S26 to 0.81 in S13. These results indicate that the diversity and composition of protease producing bacterial communities varied among different sampling sites (Table 1).

### Diversity of the extracellular proteases produced by the bacteria

The diversity of bacterial extracellular proteases evaluated through inhibitor analyses are shown in Table 2. Out of the 121 isolates, only 62 produced enough proteases for enzymatic inhibition tests. PMSF inhibited all the protease activities of all 62 isolates but with varying levels ranging from 19.36 to 100%, indicating that all the isolates produced serine proteases but in different proportions. Furthermore, the enzyme activities of 11 isolates were inhibited by PMSF by more than 90%, indicating that they mainly or only produced serine protease. The enzyme activities of 27 isolates were inhibited by OP with an efficiency ranging from 21.12 to 66.37% and slight inhibition (2.5–19.04%) in 25 isolates, while no inhibitory effect was observed in 10 isolates, suggesting that most isolates produced metalloproteases. Meanwhile, 52 out of 62 (83.87%) isolates were inhibited by both PMSF and OP at different levels, illustrating that most isolates tested in this study produced serine proteases and metalloproteases. E-64 inhibited the proteolytic activities of 7 isolates by 12.14–65.11%, and PA inhibited 7 isolates with 12.91–34.5% success. These indicate that around 11% of the isolates produced cysteine and/or aspartic proteases. Therefore, nearly all the extracellular proteases excreted by the bacteria isolated from the sediment samples belonged to serine protease and/or metalloproteases, and only a small portion produced cysteine proteases or/and aspartic proteases.

**Table 2:** **Summary of the diversity analysis of the extracellular proteases of the screened strains isolated from Laizhou Bay**.

**Genera**	**Strains**	**H/C ratio^a^**			**Inhibition ratio^c^ (%)**			
		**Casein**	**Gelatin**	**Elastin**	**PMSF (1 mM)**	**OP (1 mM)**	**E64 (0.1 mM)**	**P-A (0.1 mM)**
*Bacillus*	7-1	1.52	2.70	2.22	93.73	51.64	32.16	0.96
	7-2	2.15	5.20	0	43.83	3.09	0	0
	7-3	2.28	5.50	2.33	66.43	21.12	1.53	0
	7-5	1.55	9.80	5.20	75.98	21.63	0	4.37
	7-7	3.25	8.00	3.00	94.16	66.37	12.66	1.67
	7-12	2.71	5.00	0	51.94	36.47	0	22.59
	8-6	1.78	2.39	0	42.89	0	0.44	0
	8-17	2.00	6.00	2.00	50.90	10.46	0	0
	16-3	2.17	6.00	0	89.80	25.23	0	0
	16-10	2.57	5.25	3.25	47.54	8.98	1.85	7.87
	22-2	3.33	3.77	0	92.38	8.94	0	1.37
	22-4	3.00	4.00	2.00	61.70	41.27	0	0
	22-10	2.33	7.00	0	55.77	65.14	0	0
	22-11	4.00	7.25	0	104.11	5.65	0	1.84
	22-12	2.18	4.09	0	97.53	33.82	8.20	0
	22-17	3.00	5.75	0	19.36	47.90	0	5.13
	22-21	5.33	7.00	0	23.64	43.01	0	0
	26-3	2.15	3.80	5.00	91.55	17.08	0	0
	26-4	2.25	3.36	0	56.83	41.55	0	0
	26-7	2.45	3.50	2.17	33.83	28.98	3.51	12.91
	26-9	3.00	5.75	0	77.68	18.24	0	0
	26-10	2.33	6.20	2.60	95.57	42.40	0	0
	26-12	1.67	7.25	5.50	46.70	14.22	0	0
	26-13	1.33	1.33	0	86.92	11.16	0	5.52
	26-14	2.17	4.29	3.83	91.82	54.56	0	14.19
	26-15	3.43	4.55	1.45	56.25	5.62	1.66	0
	26-17	1.33	5.50	0	37.57	30.61	13.04	0
	26-18	3.67	8.20	2.33	99.01	25.94	0	5.43
	26-19	1.60	2.46	3.17	58.23	13.54	5.71	0
	26-22	3.30	4.29	Thin^b^	95.02	56.21	0	6.49
	70054	3.43	1.25	0	46.13	13.09	0	0
*Jeotgalibacillus*	22-7	1.25	1.33	0	47.07	24.63	5.86	0
*Planococcus*	26-20	3.88	8.50	3.17	48.40	13.52	0	0
*Oceanobacillus*	26-6	2.00	2.67	0	59.03	16.62	7.32	0
*Rheinheimera*	16-9	5.00	5.71	3.29	55.93	10.62	4.97	0
*Celeribacter*	70070	3.00	3.11	0	80.49	9.29	2.74	12.96
*Pseudoalteromonas*	7-4	2.12	2.16	0	65.55	30.12	0	0
	7-13	3.54	3.00	0	67.03	0	0	34.50
	7-15	2.47	2.27	1.17	66.51	15.53	0	0
	8-3	1.90	1.81	1.25	65.86	27.70	0	0
	8-12	2.92	2.47	0	61.20	42.12	0	0
	8-13	2.93	2.39	1.36	69.55	36.86	0	0
	8-19	2.71	2.50	Thin	64.75	17.21	0	0
	13-18	2.71	4.00	1.65	94.07	35.94	0	0
	16-4	2.40	5.00	0	42.16	6.54	0	0
	16-5	2.36	4.71	1.31	83.87	35.05	0	0
	16-11	2.25	4.57	0	41.52	0	0	0
	16-12	2.60	4.43	0	70.85	0	12.14	31.84
	26-5	3.20	1.84	2.50	64.99	29.63	12.65	0
	70021	2.31	2.30	0	33.57	0	0	0
	70317	2.94	2.15	1.33	66.71	16.20	2.78	0
	70340	3.60	1.20	2.09	10.33	19.04	0	0
	70357	2.63	1.50	2.33	54.87	2.50	0	18.26
	70363	3.00	2.75	1.67	44.61	0	28.61	0
*Photobacterium*	13-4	5.29	4.30	0	67.94	0	0	0
	13-5	4.00	4.00	0	71.79	11.13	5.32	0
	13-12	3.00	3.18	0	66.96	0	0	0
	13-13	3.50	4.71	0	78.18	7.26	0	5.90
	13-16	3.89	2.00	0	64.21	0	0	0
	13-20	3.14	1.33	0	66.97	14.00	65.11	0
*Micrococcus*	70016	1.75	2.09	0	76.53	0	0	0
*Nocardioides*	70071	2.63	4.00	0	23.58	43.78	4.43	0

a *H/C ratio is the ratio of the hydrolytic zone diameter vs. the colony diameter of a colony on the plate*.

b *Thin represents a slight hydrolytic zone formed by a single colony*.

c *Inhibition ratio (%) was calculated by using control activity minus the relative activity of a sample with an inhibitor and the activity of a sample without any inhibitor was taken as a control (%)*.

*PMSF, phenylmethylsulfonyl fluoride; OP, 1, 10-phenanthroline; P-A, pepstatin A*.

The protease diversity of all the bacteria isolated from Laizhou Bay sediments was also evaluated through their hydrolytic abilities (H/C ratio) against different types of proteins including casein, gelatin and elastin supplemented on solid media. As shown in Table 2 (also Table S2), most of the isolates formed apparent hydrolytic zones on the plates containing casein or gelatin, except 22-14, 70019, 70406, 70422, and 70423, which could not hydrolyze casein, and 7-6, 22-6, 70017, 70018, and 70334 that were not capable of hydrolyzing gelatin. Isolates belonging to *Bacillus* 22-21, *Photobacterium* (13-4) and *Rheinheimera* (16-9) showed high caseinolytic activity with H/C ratio greater than 5.0, and isolates of *Bacillus* (7-2, 7-3, 7-5, 7-7, 7-12, 8-17, 16-3, 16-10, 22-10, 22-11, 22-17, 22-21, 26-9, 26-10, 26-12, 26-17, and 26-18), *Planococcus* (26-20), *Rheinheimera* (16-9) and *Pseudoalteromonas* (16-4) exhibited strong gelatinolytic activity with H/C ratio greater than 5.0, in which the isolate 7-5 presented the highest H/C ratio 9.8 on gelatin plate. However, only 42 isolates (34.71% of the total isolates) possessed the ability to hydrolyze elastin. *Bacillus* isolates 26-12, 7-5, and 26-3 showed the highest elastinolytic activity with H/C ratio greater than 5.0. All elastinolytic isolates also showed caseinolytic and gelatinolytic activities, especially *Bacillus* strains 26-12 and 7-5 that actively degraded both elastin and gelatin. In general, the *Bacillus* isolates showed higher caseinolytic, gelatinolytic and elastinolytic activities than the other isolates from the other genera in this study. Furthermore, varied levels of degradation were observed among the isolates, even for isolates belonging to the same genus.

## Discussion

Protease-producing bacteria play essential roles in the decomposition and recycling of organic nitrogen in marine ecosystems but knowledge on their diversity information is rare, especially in China's coastal environments (Zhang et al., 2015). In this study, the phylogenetic diversity of cultivable protease-producing bacteria isolated from the sediments of Laizhou Bay, China, and the diversity of the extracellular proteases secreted by these bacteria were investigated.

This is the first report focused on protease-producing bacteria in Laizhou Bay, the most abundant of which was found to have 10^−4^ CFU g^−1^ in the seven sediment samples, which was similar to the community of protease producing bacteria isolated in Jiaozhou Bay (Zhang et al., 2015) but lower than the isolates from South China Sea (10^6^ cells/g) and sub-Antarctic sediments (10^5^ cells/g) (Zhou et al., 2009, 2013). The 121 isolates belonged to 17 genera from four phyla. Compared with previous studies (Zhou et al., 2009, 2013; Zhang et al., 2015), this is the first time that bacteria belonging to *Jeotgalibacillus, Oceanobacillus, Sulfitobacter, Celeribacter, Ruegeria, Alcanivorax, Nocardioides*, and *Salegentibacter* were reported to possess protease-producing abilities. Bacterial diversity has been rarely investigated in Laizhou Bay, and a recent investigation based on culture-independent pyrosequencing (FLX 454) method revealed extremely diverse communities in the sediments of Laizhou Bay (Wang et al., 2014). The authors identified at least 36 phyla with *Proteobacteria* (>40%) as the dominant phylum and *Gammaproteobacteria* as the dominant class. *Gammaproteobacteria* is an important class that has universal distribution in marine sediments (Polymenakou et al., 2005; Hunter et al., 2006; Xu et al., 2008). Similarly, in our study, *Proteobacteria* was the most abundant cultivable protease-producing bacterial phyla (54.7%) with *Gammaproteobacteria* as the dominating class (50.4%) (Table 1, Table S1, and Figure 2). This result is consistent with previous findings that *Gammaproteobacteria* was the predominant cultivable protease-producing bacteria in sediments, also reported in South China Sea, Jiaozhou Bay in China and sub-Antarctic samples (Olivera et al., 2007; Zhou et al., 2009; Zhang et al., 2015). This suggests that *Gammaproteobacteria* might be one the main protease-producing bacteria widely distributed in different marine environments. Phylogenetically, the 61 *Gammaproteobacteria* isolates were subdivided into different genera including *Pseudoalteromonas, Photobacterium, Marinobacter, Halomonas, Alcanivorax*, and *Rheinheimera*, in which *Pseudoalteromonas* (49 strains, accounting for 80.3% of all the *Gammaproteobacteria* strains) was dominant and widespread, being found in all seven samples.

*Firmicutes* was the second most abundant phylum, with *Bacillus* as the dominant genus (Table 1 and Figure 2). This was consistent with reports using culture-independent methods (Zhu et al., 2013), demonstrating that *Firmicutes* or *Bacillus* might play important roles in biodegradation of coastal sediments and warrant further studies. Furthermore, *Bacillus* strains are widely distributed and could easily adapt in terrestrial and coastal environments, with some presumed to have originated from terrestrial environments and became adapted to marine conditions. Consistent with this was the presence of protease-producing *Bacillus* being dominant in both Jiaozhou Bay and sub-Antarctic coastal sediment coastal sediment samples (Zhou et al., 2013; Zhang et al., 2015).

As a semi-enclosed bay, Laizhou Bay is a traditional area for aquaculture (e.g., fish, shrimp, trepan and crab). *Bacillus* could degrade wastes from these farms such as shrimp shell (Sorokulova et al., 2009), while *Pseudoalteromonas* and *Photobacterium* were reported to be associated with marine animals such as fish (Belchior and Vacca, 2006; Urbanczyk et al., 2011). The high proportions of *Pseudoalteromonas* and *Bacillus* in sediments of Laizhou Bay imply that they maybe interacting with marine animals or could also be degrading wastes from fisheries. In addition, they could regulate proliferation of other species through the production of inhibitory/antibiotic secondary metabolites, thus, also influencing the overall structure of the community (Bowman, 2007; Mondol et al., 2013).

Although species composition or abundance differed among sites, no significant correlations were detected between bacterial diversity indices or composition and the OrgC and OrgN levels or C/N ratios. However, abundance of the dominant protease-producing bacterial species seemed to vary with the depth of the sampling sites except in S8. For example, *Bacillus* was found to be more abundant in the shallower sampling sites (S7, S22, and S26) where the depth was less than 10.5 m but were not found in the deeper sites using high throughout sequencing (Zinger et al., 2011). The abundant *Bacillus* in the sediments of Laizhou Bay located near China's second longest river, the Yellow River's estuary, could have terrestrial origins (Zhang et al., 2015), and could partially explain why these bacteria were dominant in the shallow sea sediment samples. In contrast, *Pseudoalteromonas* only dominated in sediments deeper than 11.4 m, particularly from sites S5, S13, and S16 in this study. It seems that *Pseudoalteromonas* was more suitable to propagate in deeper environments.

Similar to studies in South China Sea (Zhou et al., 2009), sub-Antarctic coastal sediments (Zhou et al., 2013) and Jiaozhou Bay, China (Zhang et al., 2015), strains isolated in this study mainly produced serine and/or metallo-proteases. Also, among the three protein substrates tested in this study, elastin was the most insoluble due to its high molecular protein polymer, and thus the most difficult to degrade. This resulted to only a small portion of isolates exhibiting degradation activity. Although some isolates possessed identical 16S rRNA gene sequences, indicating same species such as isolates 26-4 and 26-19, 7-1, and 22-2 belonging to *Bacillus* or 8-10, 22-6, 22-14, and 26-21 belonging to *Halobacillus*, they still showed different levels of degradation activities for different substrates. Thus, the high enzymatic strain selection should not just select representative strains according to taxonomy but should include screening of each strain. Furthermore, the higher proteolytic activities for casein, gelatin and/or elastin of *Bacillus* isolates in this study differed from the protease-producing bacteria from those in South China Sea, which indicate that *Bacillus* isolates possess low protease-producing abilities (Zhou et al., 2009). Strains with different enzyme types (e.g., inhibited by different inhibitors such as E64, P-A) and possessing different specificity for substrates were observed in the same sample (Table 2 and Table S2) as suggested by Obayashi and Suzuki (2008), which may allow the bacterial community to more effectively hydrolyze diverse and complex organic nitrogen present in the sediments of Laizhou Bay.

In conclusion, this study investigated the community structure of the cultivable extracellular protease-producing bacteria and their protease types in sediments of Laizhou Bay. *Bacillus, Pseudoalteromonas* and *Photobacterium* dominated the communities with serine- and/or metallo-proteases as the major extracellular proteases secreted by these bacteria. In addition, most strains showed high activities in degrading casein and/or gelatin but only small proportion showed elastinolytic activity. As the first systematic study that investigated the protease-producing bacteria in Laizhou Bay even in Bohai Sea, our study provided new insights on protease-producing bacteria and their extracellular protease diversity. Furthermore, some candidate novel bacteria showed high protease degradation activities. This is also the first time that species belonging to *Jeotgalibacillus, Oceanobacillus, Sulfitobacter, Celeribacter, Ruegeria, Alcanivorax, Nocardioides*, and *Salegentibacter* were reported to possess proteolytic activities, implying that sediment bacteria may be a reservoir for novel proteases, as well as for novel bacteria.

## Author contributions

YL responsible for setting all the experiment, bacterial isolation, enzyme activity test and prepare the manuscript. CW determined all the enzyme activities and ihibiton test. MZ isolated most of the bacteria isolates. EW revised the manuscript and produced constructive suggestions. ZZ and WL participate in part of the all experiment. JN depicted the figures for this paper. ZX responsible for the experiment setting and the funding support.

### Conflict of interest statement

The authors declare that the research was conducted in the absence of any commercial or financial relationships that could be construed as a potential conflict of interest.
