# Supplementary material for: Diversity of Cultivable Protease-Producing Bacteria in Laizhou Bay Sediments, Bohai Sea, China
Source: Front Microbiol. 2017 Mar 16;8:405. doi: 10.3389/fmicb.2017.00405 (PMC5352678; doi:10.3389/fmicb.2017.00405)
Supplement: Supplementary file 1 [file Table1.PDF]

Table S1 Distribution and taxonomy affiliation of protease producing bacteria.

Phyla (Strains number)	Genera (Strains number)	Stations						
		S5	S7	S8	S13	S16	S22	S26
Firmicutes (53)	*Bacillus* **(44)**		7-1, 7-2, 7-3, 7-5, 7-6, 7-7, 7-9, 7-12, 70018, 70054	8-6, 8-15, 8-17		16-3, 16-10	22-1, 22-2 22-3, 22-4, 22-8, 22-9, 22-10, 22-11, 22-12, 22-13, 22-15, 22-17, 22-20, 22-21	26-3, 26-4, 26-7, 26-9, 26-10, 26-11, 26-12, 26-13, 26-14, 26-15, 26-16, 26-17, 26-18, 26-19, 26-22
	Jeotgalibacillus **(1)**						22-7	
	Planococcus **(1)**							26-20
	Oceanobacillus **(2)**							26-6, 26-8
	Halobacillus **(5)**			8-10			22-5, 22-6, 22-14	26-21
Proteobacteria (67)	*Pseudoalteromonas* **(49)**	70311, 70314, 70316, 70317, 70318, 70334, 70340, 70353, 70357, 70363, 70410	7-4, 7-8, 7-13, 7-14, 7-15, 70017	8-2, 8-3, 8-4, 8-5, 8-7, 8-8, 8-9, 8-11, 8-12, 8-13, 8-16, 8-18, 8-19	13-14, 13-17, 13-18	16-1, 6-2, 16-4, 16-5, 16-6, 16-7, 16-8, 16-11, 16-12, 70019, 70020	22-16, 22-18, 22-19, 70021	26-5
	Sulfitobacter **(1)**	70406						
	Marinobacter **(2)**	70422, 70423						
	Halomonas **(1)**				13-8			
	Rheinheimera **(1)**					16-9		
	Celeribacter **(1)**							70070
	Photobacterium **(7)**		7-11		13-4, 13-5, 13-12, 13-13, 13-16, 13-20			

	Ruegeria **(2)**		70076, 70077					
	Alcanivorax **(1)**						70072	
Actinobacteria (2)	*Micrococcus* **(1)**	70016						
	Nocardioides **(1)**				70071			
Bacteroidetes (1)	*Salegentibacter* **(1)**	70409						
Total	121	16	19	17	11	14	23	21

Table S2 The H/C ratio of all the strains isolated in this study grown on the plates containing casein, gelatin, elastin respectively.

Genera	Strains	H/C ratio[a]			Genera	Strains	H/C ratio		
		Casein	Gelatin	Elastin			Casein	Gelatin	Elastin
Bacillus	7-1	1.52	2.70	2.22	*Pseudoalteromonas*	7-4	2.12	2.16	0
	7-2	2.15	5.20	0		7-8	2.40	2.25	0
	7-3	2.28	5.50	2.33		7-13	3.54	3.00	0
	7-5	1.55	9.80	5.20		7-14	2.47	2.88	1.45
	7-6	4.20	Thin	0		7-15	2.47	2.27	1.17
	7-7	3.25	8.00	3.00		8-2	1.91	2.10	1.45
	7-9	1.94	2.94	0		8-3	1.90	1.81	1.25
	7-12	2.71	5.00	0		8-4	2.44	2.47	0
	8-6	1.78	2.39	0		8-5	2.85	2.10	0
	8-15	1.55	2.27	0		8-7	2.35	2.61	1.27
	8-17	2.00	6.00	2.00		8-8	2.31	2.44	0
	16-3	2.17	6.00	0		8-9	2.57	3.00	0
	16-10	2.57	5.25	3.25		8-11	2.69	2.08	0
	22-1	2.89	4.30	0		8-12	2.92	2.47	0
	22-2	3.33	3.77	0		8-13	2.93	2.39	1.36
	22-3	2.50	3.75	0		8-16	2.29	2.27	0
	22-4	3.00	4.00	2.00		8-18	2.38	2.17	1.38
	22-8	1.40	2.86	0		8-19	2.71	2.50	Thin
	22-9	2.30	2.60	0		13-14	2.83	2.92	0
	22-10	2.33	7.00	0		13-17	2.71	3.10	0
	22-11	4.00	7.25	0		13-18	2.71	4.00	1.65
	22-12	2.18	4.09	0		16-1	2.06	2.19	Thin
	22-13	1.50	2.00	0		16-2	2.50	3.50	0
	22-15	2.75	4.57	0		16-4	2.40	5.00	0
	22-17	3.00	5.75	0		16-5	2.36	4.71	1.31
	22-20	2.33	4.00	0		16-6	2.18	2.67	Thin
	22-21	5.33	7.00	0		16-7	2.20	2.92	1.25
	26-3	2.15	3.80	5.00		16-8	2.33	2.62	1.36
	26-4	2.25	3.36	0		16-11	2.25	4.57	0
	26-7	2.45	3.50	2.17		16-12	2.60	4.43	0
	26-9	3.00	5.75	0		22-16	2.40	3.36	0
	26-10	2.33	6.20	2.60		22-18	2.18	3.00	0
	26-11	1.40	3.36	0		22-19	2.45	3.27	0
	26-12	1.67	7.25	5.50		26-5	3.20	1.84	2.50
	26-13	1.33	1.33	0		70017	1.44	0	0
	26-14	2.17	4.29	3.83		70019	0	1.60	0
	26-15	3.43	4.55	1.45		70020	1.50	3.33	0
	26-16	1.80	3.20	0		70021	2.31	2.30	0
	26-17	1.33	5.50	0		70311	3.13	1.30	1.55

	26-18	3.67	8.20	2.33		70314	2.64	3.00	1.64
	26-19	1.60	2.46	3.17		70316	2.94	2.64	1.36
	26-22	3.30	4.29	Thin[b]		70317	2.94	2.15	1.33
	70018	1.38	0	0		70318	1.78	2.18	0
	70054	3.43	1.25	0		70334	2.36	0	1.40
Jeotgalibacillus	22-7	1.25	1.33	0		70340	3.60	1.20	2.09
Planococcus	26-20	3.88	8.50	3.17		70353	3.58	3.00	1.70
Oceanobacillus	26-6	2.00	2.67	0		70357	2.63	1.50	2.33
	26-8	1.60	2.00	0		70363	3.00	2.75	1.67
Halobacillus	8-10	2.83	2.65	1.17		70410	2.81	1.17	1.58
	22-5	4.67	3.83	0	*Photobacterium*	7-11	1.91	3.89	0
	22-6	1.17	Thin	0		13-4	5.29	4.30	0
	22-14	0	2.83	0		13-5	4.00	4.00	0
	26-21	2.20	3.00	0		13-12	3.00	3.18	0
Sulfitobacter	70406	0	1.33	0		13-13	3.50	4.71	0
Marinobacter	70422	0	1.25	0		13-16	3.89	2.00	0
	70423	0	1.33	0		13-20	3.14	1.33	0
Halomonas	13-8	1.29	1.25	0	*Micrococcus*	70016	1.75	2.09	0
Rheinheimera	16-9	5.00	5.71	3.29	*Nocardioides*	70071	2.63	4.00	0
Celeribacter	70070	3.00	3.11	0	*Ruegeria*	70076	1.75	2.75	0
Salegentibacter	70409	1.60	3.20	2.17		70077	2.17	2.82	0
Alcanivorax	70072	1.50	3.50	0					

[a] H/C ratio is the ratio of the hydrolytic zone diameter versus the colony diameter of a colony on the plate

[b] Thin represents a slight hydrolytic zone formed by a single colony
