# Supplementary material for: Diversity of Cultivable Protease-Producing Bacteria in Laizhou Bay Sediments, Bohai Sea, China
Source: Front Microbiol. 2017 Mar 16;8:405. doi: 10.3389/fmicb.2017.00405 (PMC5352678; doi:10.3389/fmicb.2017.00405)
Supplement: Supplementary file 2 [file Image1.PDF]

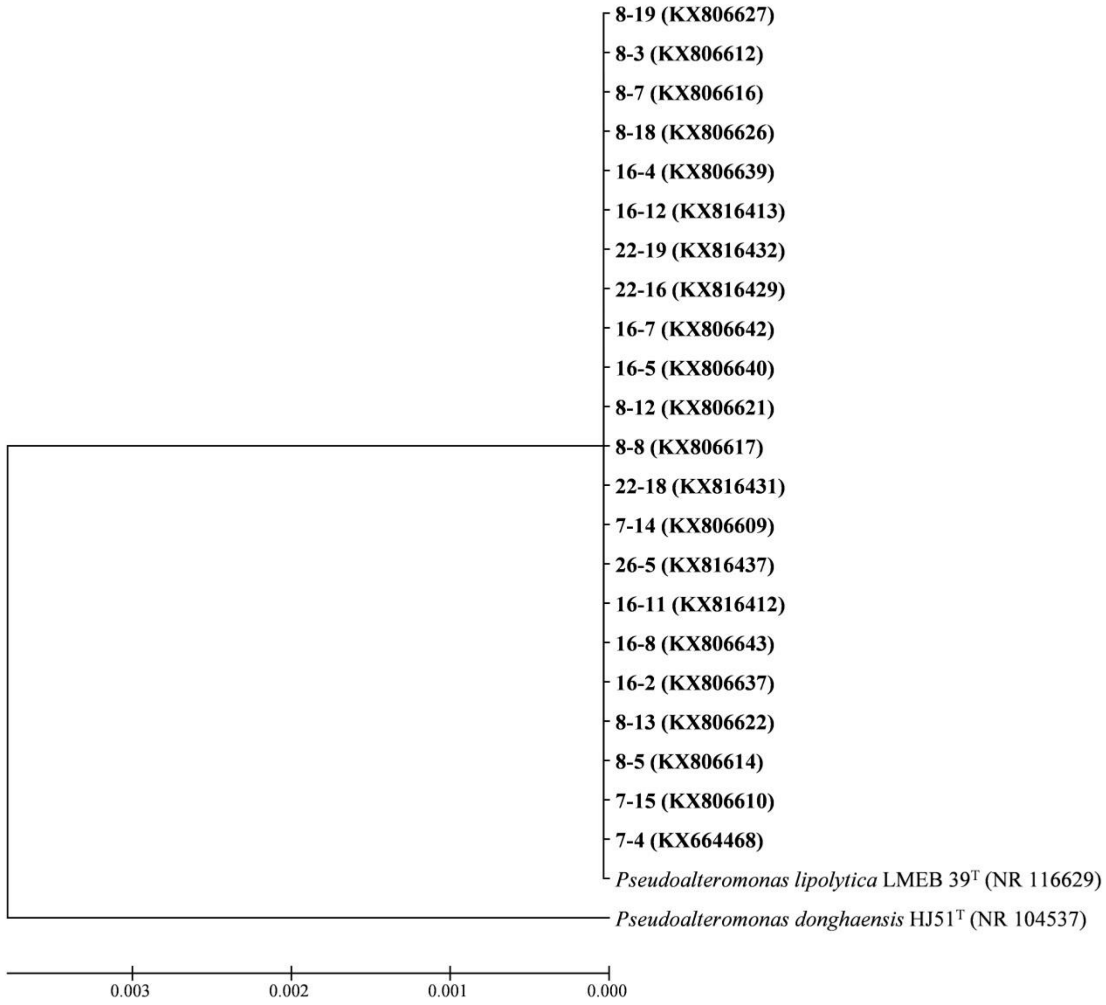

Figure S1 The Neighbor-joining phylogenetic tree of the strains in Branch 1 in Figure 3 based on the 16S rRNA gene sequences.

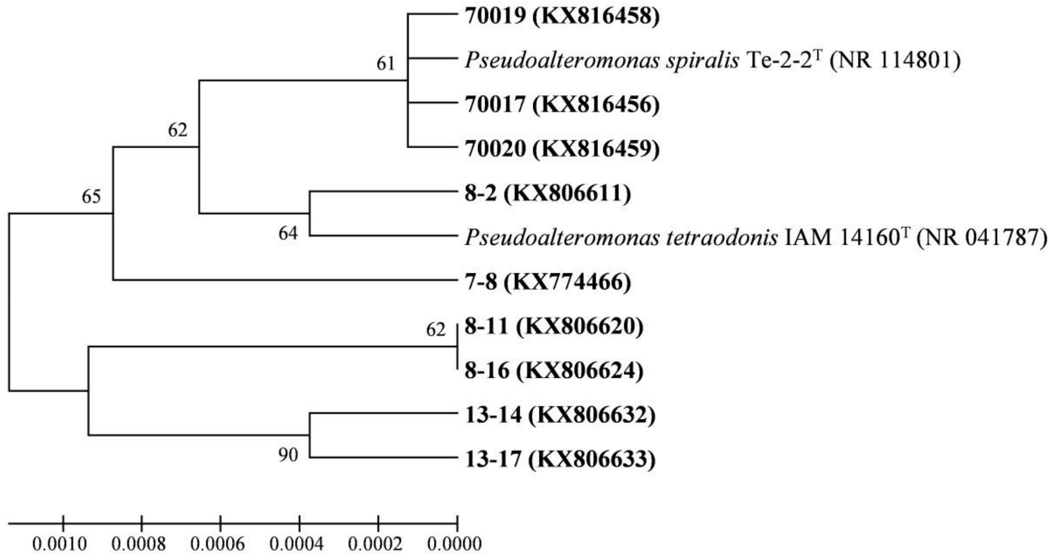

Figure S2 The Neighbor-joining phylogenetic tree of the strains in Branch 2 in Figure 3 based on the 16S rRNA gene sequences.

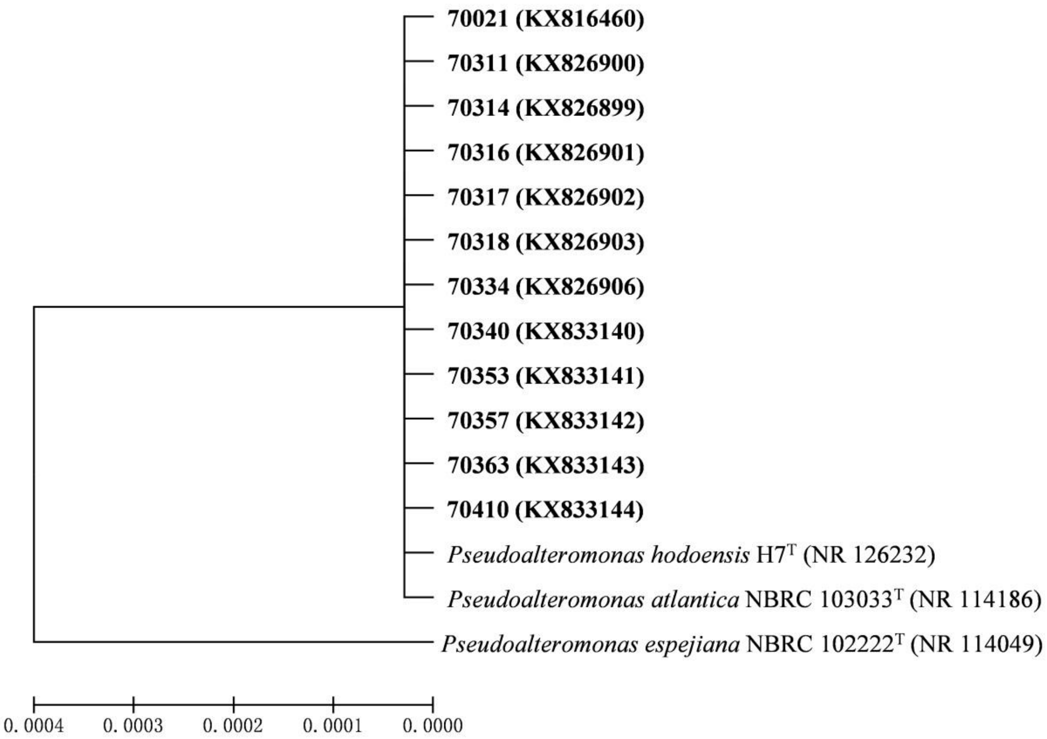

Figure S3 The Neighbor-joining phylogenetic tree of the strains in Branch 3 in Figure 3 based on the 16S rRNA gene sequences.
